# Mechanical strength assessment of a drilled hole in the contralateral cortex at the end of the open wedge for high tibial osteotomy

**DOI:** 10.1186/s40634-017-0098-0

**Published:** 2017-06-23

**Authors:** Arnaud Diffo Kaze, Stefan Maas, Alexander Hoffmann, Dietrich Pape

**Affiliations:** 1 Faculty of Science, Technology and Communication, University of Luxembourg, 6, rue R. Coudenhove-Kalergi, L-1359 Luxembourg, Luxembourg; 2 Department of Orthopedic Surgery, Centre Hospitalier de Luxembourg, L-1460 Luxembourg, Luxembourg; 3 Sports Medicine Research Laboratory, Public Research Centre for Health, Luxembourg, Centre Médical de la Fondation Norbert Metz, 76 rue d’Eich, L-1460 Luxembourg, Luxembourg; 4 Cartilage Net of the Greater Region, 66421 Homburg/Saar, Germany

**Keywords:** High tibial osteotomy (HTO), Osteoarthritis (OA), Opposite cortical fracture, Drill hole, Correction angle, Biomechanics, Finite element analysis, Static strength, Elongation at break

## Abstract

**Background:**

This study aimed to investigate, by means of finite element analysis, the effect of a drill hole at the end of a horizontal osteotomy to reduce the risk of lateral cortex fracture while performing an opening wedge high tibial osteotomy (OWHTO). The question was whether drilling a hole relieves stress and increases the maximum correction angle without fracture of the lateral cortex depending on the ductility of the cortical bone.

**Methods:**

Two different types of osteotomy cuts were considered; one with a drill hole (diameter 5 mm) and the other without the hole. The drill holes were located about 20 mm distally to the tibial plateau and 6 mm medially to the lateral cortex, such that the minimal thickness of the contralateral cortical bone was 5 mm. Based on finite element calculations, two approaches were used to compare the two types of osteotomy cuts considered: (1) Assessing the static strength using local stresses following the idea of the FKM-guideline, subsequently referred to as the “FKM approach” and (2) limiting the total strain during the opening of the osteotomy wedge, subsequently referred to as “strain approach”. A critical opening angle leading to crack initiation in the opposite lateral cortex was determined for each approach and was defined as comparative parameter. The relation to bone aging was investigated by considering the material parameters of cortical bones from young and old subjects.

**Results:**

The maximum equivalent (von-Mises) stress was smaller for the cases with a drill hole at the end of the osteotomy cut. The critical angle was approximately 1.5 times higher for the specimens with a drill hole compared to those without. This corresponds to an average increase of 50%. The calculated critical angle for all approaches is below 5°. The critical angle depends on the used approach, on patient’s age and assumed ductility of the cortical bone.

**Conclusions:**

Drilling a hole at the end of the osteotomy reduces the stresses in the lateral cortex and increases the critical opening angle prior to cracking of the opposite cortex in specimen with small correction angles. But the difference from having a drill hole or not is not so significant, especially for older patients. The ductility of the cortical bone is the decisive parameter for the critical opening angle.

## Background

Valgus-producing high tibial osteotomy is recognized as an effective surgical procedure for the treatment of unicompartmental osteoarthritis (OA) of the knee in the presence of axial malalignment in active patients. Precise preoperative planning (Pape et al. 2004a, 2004b) and a high primary stability of the implant-bone-construct are required for a good result. An intact lateral cortical hinge is important for the stability of an opening wedge high tibial osteotomy (OWHTO) because fractures have been reported to cause loss of correction and poor clinical results (Agneskirchner et al. 2006; Stoffel et al. 2004; van Raaij et al. 2008; Türkmen et al. 2016; Jo et al. 2017). In order to prevent the fracture of the medial cortical hinge while performing closing wedge high tibial osteotomy (CWHTO), Kessler et al. terminated the osteotomy cut with a 5-mm diameter bored hole in the anterior-posterior (AP) direction; which allowed a significant increase of the correction angle compared with the same osteotomy without a drill hole (Kessler et al. 2002). The present study aimed to investigate mechanically the effect of such a potentially stress relieving drill hole in the AP direction at the end of the horizontal osteotomy cut while performing an OWHTO by comparing the static strength of the lateral cortical bone in cases of osteotomies with or without a drill hole. Two approaches were used to achieve this objective: (1) Assessing the static strength using local stresses following the ideas of the FKM guidelines “Analytical Strength Assessment of Components”, commonly known as the FKM guidelines (Rennert et al. 2012), and (2) limiting the total strain to the elongation at break during the opening of the osteotomy wedge. FKM is an abbreviation of the german expression "Forschungskuratorium Maschinenbau" (Rennert et al. 2012). The first approach was designated “FKM approach” and the second “strain approach”. Both approaches rely on non-linear finite element analyses. The assessment was based on crack initiation only, hence complete rupture and dislocation was not investigated here. To our knowledge, no study investigated so far the mechanical effect of a drill hole in medial HTO. Does a drill hole at the end of the osteotomy cut increase the correction angle without fracture of the lateral cortical hinge in high tibial osteotomy? It was hypothesised that in spite of drilling a hole and significant stress relief crack initiation takes place between 2 and 5° depending on the bone ductility.

## Methods

### Specimens

**Fig. 1:**
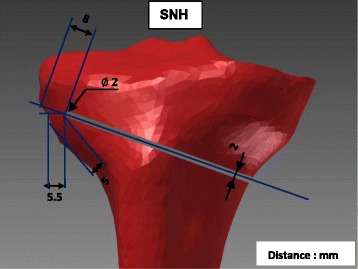
Specimen SNH: This specimen has no hole at the lateral end of the osteotomy cut, which started on the medial side at 35 mm distally to the tibial plateau and finished 6 mm from the lateral cortex, 20 mm distally to the tibial plateau. The osteotomy cut had a thickness of 2 mm and the minimal thickness of the contralateral cortical bone was 5 mm

**Fig. 2:**
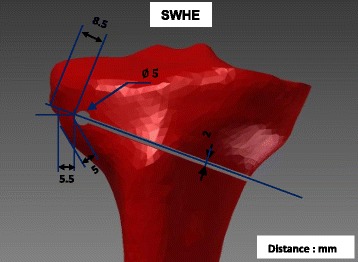
Specimen SWHE: Specimen with the drilled hole as an extension of the osteotomy cut. The osteotomy cut was realised as for specimen SNH (Fig. 1) and the drill hole of 5 mm diameter was located about 20 mm distally to the tibial plateau and 6 mm medially to the lateral cortex

**Fig. 3:**
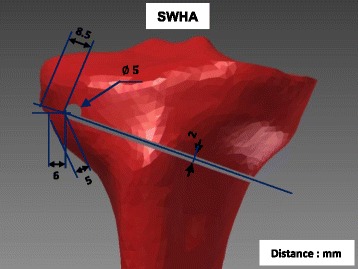
Specimen SWHA: Specimen with the drilled hole as an extension of the osteotomy cut, but above the lateral end of it. The osteotomy cut was realised as for specimen SNH (Fig. 1) and the drill hole as for specimen SWHE (Fig. 1), butwas located about 2 mm proximally

Two different types of osteotomy cuts were considered; one with a drill hole (diameter 5 mm) and the other without the hole. The osteotomy cut had a thickness of 2 mm. The starting point of the horizontal osteotomy was located on the medial side at 35 mm distally to the tibia plateau and the end point was located in the lateral cortical bone at 20 mm distally to the tibia plateau and 6 mm from the lateral cortex. The minimal thickness of the contralateral cortical bone was 5 mm. Two types of drill holes were considered: one directly at the end of the osteotomy cut as an extension and the second also at the end of the osteotomy, but above it. The drill holes were located about 20 mm distally to the tibial plateau and 6 mm medially to the lateral cortex. The specimen without hole or “Specimen with No Hole” has been designated by the abbreviation SNH (Fig. 1). The “Specimen With the Hole as an Extension” of the osteotomy cut has been called SWHE (Fig. 2) while the second “With the Hole located Above” of the osteotomy cut has been designated SWHA (Fig. 3).

The software HyperMesh (Altair Engineering, Inc., Antony, France) was used to generate the 3D geometries of the specimens from the mesh of the finite element model of the Lower Limb Model for Safety (Beillas et al. 2001). This mesh was developed using the state-of-art procedure of 3D geometries acquisition from medical computer tomography (CT) scanning and magnetic resonance imaging (MRI) data collected on a subject close to a 50th percentile male. The obtained geometry of the tibia was then imported in Autodesk Inventor (Autodesk, Inc., Neuchâtel, Switzerland) to model the osteotomy cut and the drilled hole. The finite element calculations were performed with Ansys Workbench (Ansys, Inc., Canonsburg, Pennsylvania, U.S.A).

All three specimens were designed with the same minimal thickness of the contralateral cortical of exactly 5 mm in order to keep the highly stressed material volume identical or at least as comparable as possible. The specimen SWHA has been compared to the specimen SWHE in order to check if the location of the drill hole is of importance. The drill hole of specimen SWHA was located 2.5 mm proximally to the one of specimen SWHE.

Two dimensional (2D) and three dimensional finite element analysis (FEA) were performed. For the 2D analyses, a section along the frontal plane of each of the three tibias was considered, and an offset of 10 mm from that section was made to build 2D geometries of the three specimens.

### Assessment of the static strength using local stresses: “FKM approach”

The components static strength (*σ*_*SK*_) of the lateral cortex of the tibia were assessed following the ideas of the FKM-guideline for metals (Rennert et al. 2012) though it is normally only used for steel and aluminium. Therefore some key equations are repeated subsequently.

1 $$ {\sigma}_{SK}={n}_{pl}\cdot {R}_e $$

The relevant section factor *n*_*pl*_ characterises the load bearing reserve up to a critical state of the specimen, once the elastic limit load has been exceeded by means of the permissible partial or total plasticisation of thecomponent cross-sections. *R*_*e*_ is the yield strength.

2 $$ {n}_{p l}= M I N\ \left(\sqrt{E{\varepsilon}_{e rtr}/{R}_e;{K}_p}\ \right) $$

The critical value of total strain *ε*_*ertr*_ characterises the deformation reserve and depends on the elongation at break and on the degree of multiaxiality *h* of the stresses.

3 $$ {\varepsilon}_{ertr}=\left\{\begin{array}{c}\hfill {\varepsilon}_{ref}\kern9.25em  for\kern0.5em  h\le 1/3\hfill \\ {}\hfill {\varepsilon}_0+0.3 \cdot {\left(\frac{\varepsilon_{ref} - {\varepsilon}_0}{0.3}\right)}^{3\cdot h}\kern1.5em  for\kern0.5em  h>1/3\kern0.75em \hfill \end{array}\right. $$

where *ε*_0_ = *R*_*e*_/*E* is the minimum of the critical strain at high multiaxiality and the reference strain *ε*_*ref*_ is related to the elongation at break *A*, which depends on material ductility, namely

4 $$ {\varepsilon}_{ref}=\kern0.5em \left\{\begin{array}{c}\hfill \kern.5em  A\kern3.2em  for\kern0.5em  ductile\  materials\kern3.25em \hfill \\ {}\hfill 0.4\cdot A\kern2em  for\kern0.5em  semi- ductile\  materials\kern0.75em \hfill \end{array}\right. $$

The degree of multiaxiality is calculated by means of principal stresses at the reference point, i.e. the critical point to be assessed with maximum equivalent (von-Mises) stress.

5 $$ h=\raisebox{1ex}{${\sigma}_H$}\!\left/ \!\raisebox{-1ex}{${\sigma}_V$}\right. $$

where *σ*_*H*_ = (*σ*_1_ + *σ*_2_ + *σ*_3_)/3 is the hydrostatic stress and *σ*_*V*_ the equivalent (von-Mises) stress

6 $$ {\sigma}_V = {\sigma}_{GH} = \sqrt{\frac{1}{2}\left[{\left({\sigma}_1-{\sigma}_2\right)}^2+{\left({\sigma}_2-{\sigma}_3\right)}^2+{\left({\sigma}_3-{\sigma}_1\right)}^2\right]} $$

with *σ*_1_, *σ*_2_ and *σ*_3_ being the principal stresses.

The local stresses were estimated by performing linear static FEA of opening the osteotomy cut by 10° because this value was considered as a median of the correction angles applied for HTO.

The factor *K*_*p*_ characterises the load bearing reserve from the first yielding at the reference point (the elastic limit load (ELL)) up to the plastic limit load (PLL). It is hence defined as the ratio of PLL to ELL.

7 $$ {K}_p=\raisebox{1ex}{$ PLL$}\!\left/ \!\raisebox{-1ex}{$ ELL$}\right. $$

The PLL was estimated by performing nonlinear FEA with the cortical bone as an ideal elastic–plastic material (Fig. 7). The osteotomy cut was opened until a horizontal tangent in the load–displacement diagram was reached i.e. an increasing deformation did no longer require increasing load; At this level the PLL was reached. The ELL is the load, which is achieved when the combined stress according to Eq. (8) at the reference point corresponds to the yield stress, which was determined in a linear FEA. The osteotomy cut is opened until the combined stress is equal to the yield stress.

The used failure criterion assumed that the specimen failed when the assessed static strength ( *σ*_*SK*_) exceeded the existing combined stress. Relying on the FKM guidelines, the used combined stress is based for ductile material on the von Mises yield criterion and isgiven by Eq. (6). For semi-ductile and brittle materials the combined stress is a linear combination of the von Mises stress (*σ*_*GH*_) and the combined stress based on the normal stress hypothesis (*σ*_*NH*_):

8 $$ {\sigma}_V = q \cdot {\sigma}_{NH}+\left(1- q\right) \cdot {\sigma}_{GH}\kern0.5em . $$

The weighting factor q is between 0 and 1 and depends on the ductility. Here it was chosen once as q = 0 for ductile and once as *q* = 1 for the cortical bone considered as a semi-ductile material having an elongation at break A in the same order of magnitude than grey cast iron (between 3 and 8%). The combined stress according to the normal stress hypothesis is given by

9 $$ {\sigma}_{NH}= MAX\ \left(\left|{\sigma}_1\right|; \left|{\sigma}_2\right|;\left|{\sigma}_3\right|\right)\kern0.5em . $$

**Fig. 4:**
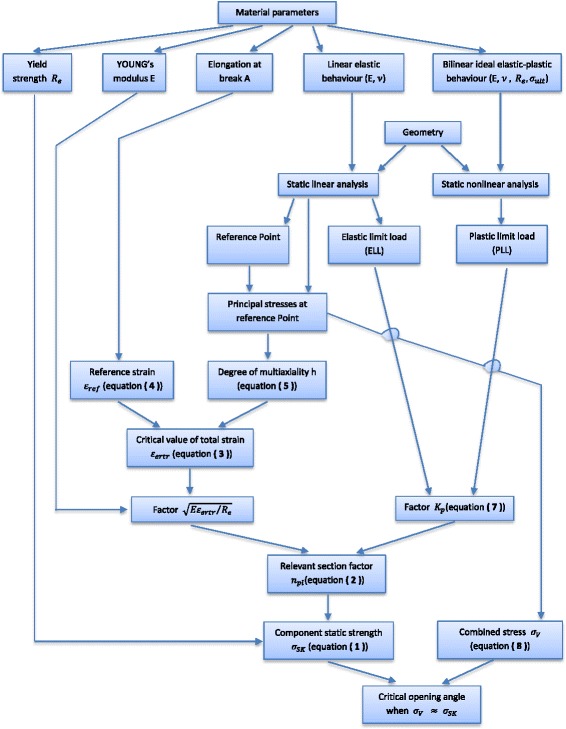
Procedure used to assess the component static stress and hence determining the critical opening angle: The critical opening angle is reached when the combined stress at the reference point reaches the component static strength

The failure load and its corresponding critical opening angle were hence reached when the existing combined stress at the reference point according to Eq. (8) reached the static strength *σ*_*SK*_ based on a linear elastic calculation. The following flowchart (Fig. 4) sketches, for a better comprehension, the procedure of the static strength assessment that has been described.

### Determination of the total strain during opening of the osteotomy wedge: “Strain approach”

The total strain of the specimen was determined by means of finite element analyses of the different specimens by considering a nonlinear behaviour for the cortical bone and opening of the osteotomy cut by 10°.

The failure criterion in case of the strain approach was based on the total strain obtained from nonlinear finite element calculations. If the calculated total strain was reaching the elongation at break A from experimental material testing, then the specimen failed. In the numerical simulation the osteotomy gap was opened and the opening angle of reaching the critical strain value (A) was hence the critical opening angle.

### Material behaviour of the cortical bone

#### Literature review

Average values of the strength properties of the cortical bone were found in the literature and are summarised in Table 1. The maximum yield (*Re*) and ultimate tensile (*σ*_*ult*_) strength of the cortical bone of the femur are 114 MPa and 135 MPa respectively, while the maximum elongation at break is *A* = *ε*_*ult*_ = 3.1% .

**Table 1:** Strength properties (average values) of the cortical bone from the literature

Authors	Specimen bone	Yield strength (*Re*) [MPa]	Ult. tensile strength (*σ*_*ult*_) [MPa]	Elongation at break (*ε*_*ult*_) [%]
(Yamada 1970)	Femur		109	1.35
(Reilly and Burstein 1975)	Femur	**114**	**135**	**3.1**
(Vinz 1975)	Femur for adults (avg. 30 years)		106	0.26
(Evans 1976)	Tibia (avg. 41.5 years)		106	1.76
(Evans 1976)	Tibia (avg. 72 years)		84	1.56
(McCalden et al. 1993) (Ott 2007, Ott et al. 2010)	Femur (avg. between [20–102] years)	71	98	2.4
(MatWeb, 2016)	Femur		135	3.1

The maximal values are in bold

Only Reilly and Burstein (1975) related ultimate shear and compression strengths to age (Table 2).

**Table 2:** Age-related strength properties of the cortical bone from (Reilly and Burstein 1975)

Age (Years)	Ultimate tensile strength *σ*_*ult*_ [MPa]			Yield strength *Re* [MPa]	Elongation at break *ε*_*ult*_ [%]
	Tension	Compression	Shear		
21		206			
22		211			
23	137		71	117	2.8
31	133	203		113	3.8
52		198			
56			65		
63	125			108	2.2
Mean values:	135	205	68	114	3.1

The values presented in the table for the different ages are the mean values obtained from the results for the tests performed on many specimens

**Fig. 5:**
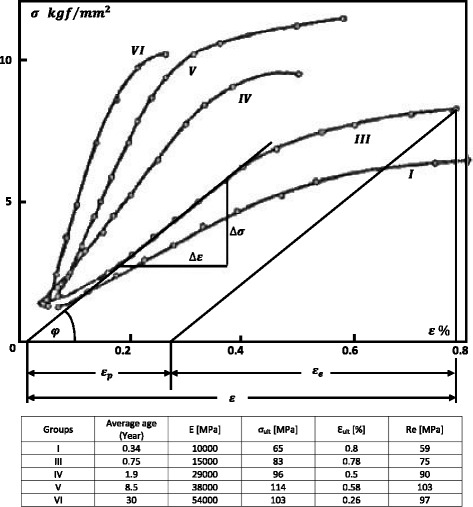
Stress–strain curves of compact bone (Vinz 1975) and the values read from the curves: The data are from seven age groups with the following average group ages in years: I) 0.05, II) 0.34, III) 0.75, IV) 1.9, V) 8.5, VI) 30 and VII) 80. The values of the elongation at break *ε*_*ult*_ from this study of H. Vinz (1975) are extremely small compared to other authors

Figure 5 shows stress–strain curves of compact bone tissue from the femur for different age groups (Vinz 1975). The values in the table near the figure were read from the curves. It should be noted that the values of the elongation at break *ε*_*ult*_ from this study (Vinz 1975) are extremely small (below 1%) compared to other authors.

**Fig. 6:**
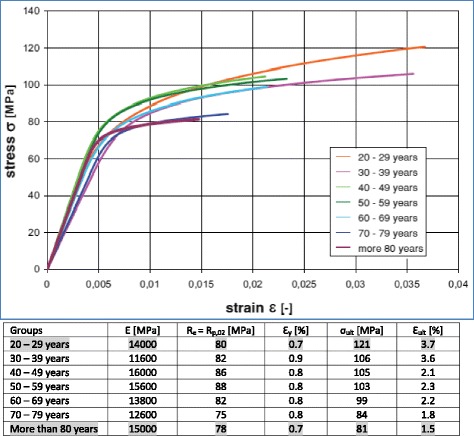
Age dependent stress–strain curves (Ott 2007; Ott et al. 2010): The values in the table were read from the curves and rounded. A 0.2% offset yield stress (*R*_*p*,0.2_) has been considered as yield stress (*R*_*e*_)

Ott et al. published more recently an age-related study of tensile properties of the cortical bone (Ott 2007; Ott et al. 2010). These authors used the experimental results published by McCalden et al. (1993) to construct age-related constitutive laws of human cortical bone (Fig. 6). The values of the strain published by Ott et al. are very large compared to the values published by Vinz in 1975 (Vinz 1975).

A study varying the patient’s age has been performed by considering the material parameters of the cortical bone of the youngest (20–29 years) and the oldest (more than 80 years) patients from the study by Ott (Ott 2007; Ott et al. 2010). The youngest (20–29 years) and oldest (more than 80 years) patients were chosen to consider a maximum variation. The mean values (Table 2) of the tensile strength properties of the cortical bone published by Reilly and Burstein (1975) have also been considered; these were the highest values found in the literature with a mean YOUNG modulus of about 17000 MPa. The study was therefore subdivided into three cases, depending on the material properties of the cortical bone. These values were considered for the 2D and 3D finite element calculations as indicated in Table 3. A Poisson’s ratio of 0.3 was used for the present study. Due to the lack of age-related material parameters of the trabecular bone, and for sake of simplicity, the tibia was considered as cortical bone only.

**Table 3:** The three different cases of the study

Case H (High)	Highest average material parameters found in the literature (mean values from Table 2)
Case M (Middle)	Material parameter of cortical bone from youngest patients (values of the group “20-29 years” from Fig. 6)
Case L (Low)	Material parameter of cortical bone from oldest patients (values of the group “more than 80 years” from Fig. 6)

The classification high, middle and low is based on the values of the yield stress

All the material parameters that were chosen to be used in the present study were retrieved from the experimental studies of Reilly and Burstein (1975) and McCalden et al. (1993). The authors of these two experimental studies stated that the bones were thawed (Reilly and Burstein 1975) or kept moist (McCalden et al. 1993) during testing.

#### Material parameters used for FKM approach

**Fig. 7:**
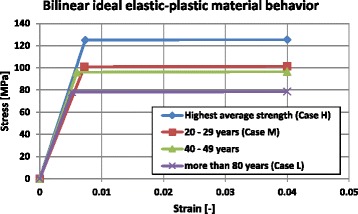
Bilinear ideal elastic–plastic curves considered to calculate the PLL: The highest average strength curve (case H) was based on the mean values of the Table 2

The stress state at the reference point with maximum combined stress (von-Mises) is decisive for the assessment of the static strength (Rennert et al. 2012). Ideal bilinear elastic–plastic stress–strain curves (Fig. 7) were used to estimate the PLL, assuming that the curves continue nearly horizontally to an infinite strain value and were not ending at 4%, as shown as example in Fig. 7. In the elastic domain the curve was defined by the Young’s modulus (E) and a fictitious yield stress taken as the mean value of the real yield stress and the ultimate stress *σ*_*y*_ = (*Re* + *σ*_*ult*_)/2. The tangent modulus has been chosen as *E*_*T*_ = 0.001 ⋅ *E* according to the FKM-guidelines (Rennert et al. 2012) and was hence nearly horizontal or ideal plastic. The maximal average strength curve was defined by simply using the mean values of the Table 2.

According to FKM and Eq. (4), *ε*_*ref*_ is different for ductile (elongation at break A ≥ 6%) and for semi-ductile material (A < 6%). The values of elongation at break A of the cortical bone from literature are smaller than 4%; this means that the cortical bone should be considered as semi-ductile according to this norm. But as the FKM-norm is conservative and addresses steel and aluminium only, hence the ductile material case was additionally investigated for reasons of comparison. The value of 6% was chosen for the elongation at break of the cortical bone considered as ductile. Therefore both material behaviours were taken into account while performing the FKM approach as indicated in Table 4.

**Table 4:** Material parameters used for FKM approach

Material parameters	Young’s modulus *E* [MPa]			Yield strength *Re* [MPa]			Elongation at break *A* [%]		
	Case H	Case M	Case L	Case H	Case M	Case L	Case H	Case M	Case L
Semi-ductile	17000	14000	15000	114	80	78	3.1%	3.7%	1.5%
Ductile	17000	14000	15000	114	80	78	6%	6%	6%

The values of the elongation at break from the literature were used for the cortical bone considered as semi-ductile, while the value of 6% was chosen for the cortical bone considered as ductile

#### Material parameters used for strain approach

**Fig. 8:**
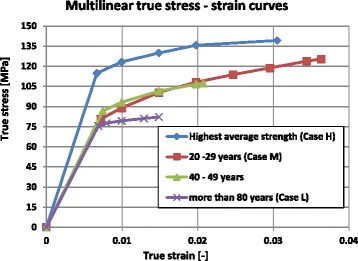
Multilinear true stress–strain curves used to evaluate the total strain: The age-related curves were based on the stress–strain curves from (Ott 2007; Ott et al. 2010). The highest average strength curve was chosen using the mean tensile strength values from (Reilly and Burstein 1975)

The multilinear true stress - strain curves (Fig. 8) were approximated by considering the values of tensile properties of the cortical bone from the study by Ott (Ott 2007; Ott et al. 2010). The highest average strength curve was defined by using the mean tensile strength values from (Reilly and Burstein 1975) by choosing points in order to reproduce the evolution of stress–strain curves of the material behaviour of the cortical bone. The true stress (*σ*_*T*_) in the tensile test is the instantaneous applied load divided by the instantaneous cross-sectional area of the considered specimen. The engineering stress or stress (*σ*) is given by the quotient of the applied load to the original cross-s ectional area of the considered specimen. The engineering strain or strain (*ε*) is the total elongation divided by the initial length of the considered specimen. The true stress ( *σ*_*T*_) is related to the stress (*σ*) by the relation *σ*_*T*_ = *σ* ⋅ (1 + *ε*) and the true strain (*ε*_*T*_) to the engineering strain (*ε*) by the relation *ε*_*T*_ = *ln*(1 + *ε*).

#### Different numerical simulations performed

2D and 3D FEA were performed. For each of analysis type, in total 27 numerical simulations were done for the 3 chosen constitutive material laws (case H, M and L), for the 3 different analysed specimens according to Figs. 1, 2 and 3, and for the 3 assumptions: strain approach, semi-ductility and ductility for FKM approach according to FKM-guideline.

The mesh of the 2D models consisted of quadrilateral elements with a size of 0.5 mm. The 3D models were meshed with tetrahedral solid elements. The mesh size of the 3D models depended on the material behaviours that were considered. For linear analyses a fine mesh size varying between 0.2 and 2 mm was used in the vicinity of the hole at the end of the osteotomy open wedge, and a mesh size varying between 2 and 5 mm was used to mesh the rest of the tibia. For nonlinear analyses the mesh size of the region in the vicinity of the hole at the end of the osteotomy open wedge was 1 mm, and the mesh size of the rest of the tibia varied between 1 and 5 mm.

## Results

### Results following the FKM approach

#### Reference point and principal stresses

**Fig. 9:**
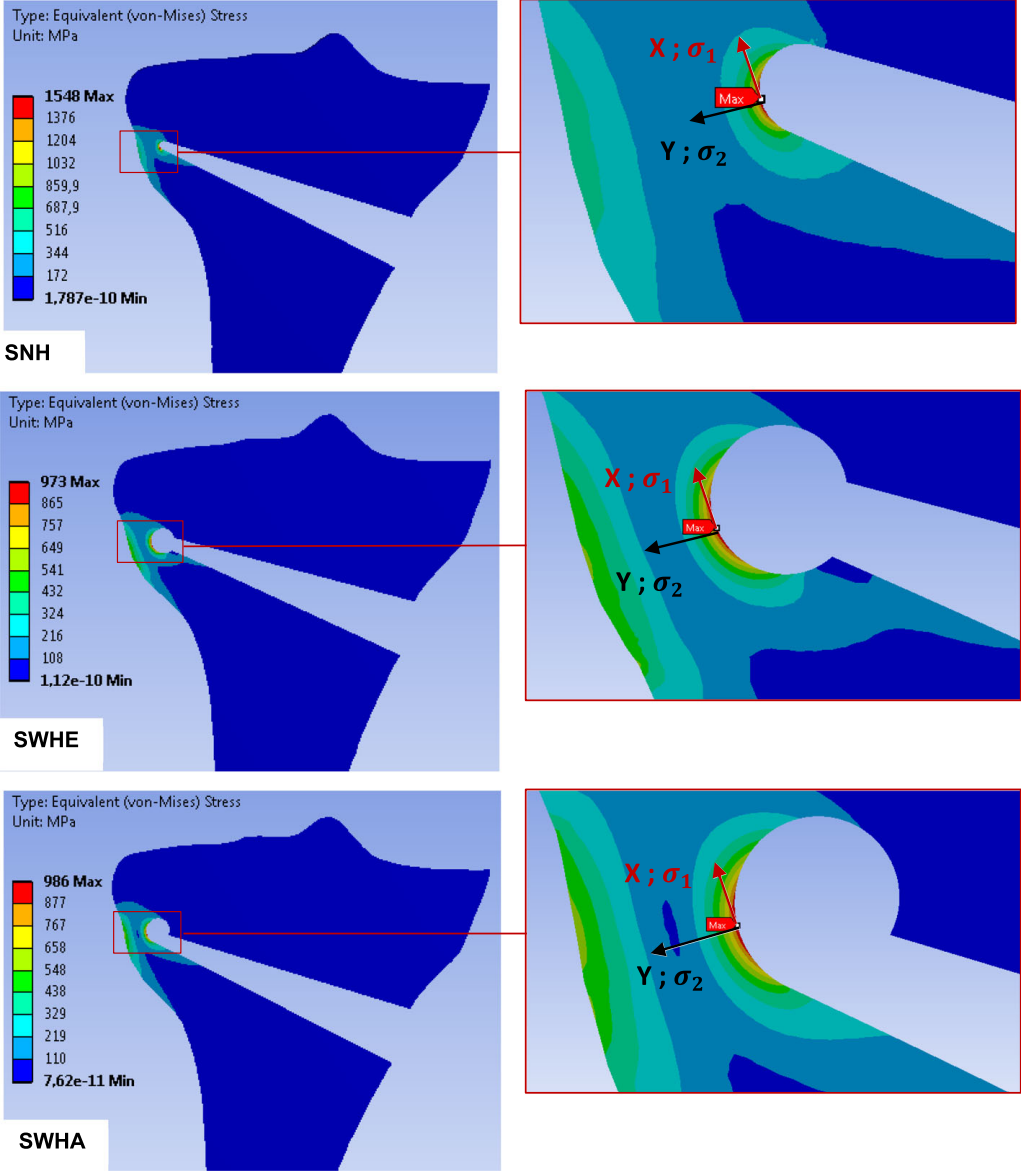
Equivalent (von-Mises) stresses obtained with 2D linear FEA: The results represented here are for the case study H. The red hotspots with the maximum equivalent (von-Mises) stress indicate the locations of the reference points as illustrated on the zoom views. The stress values correspond to an opening angle of 10°

**Fig. 10:**
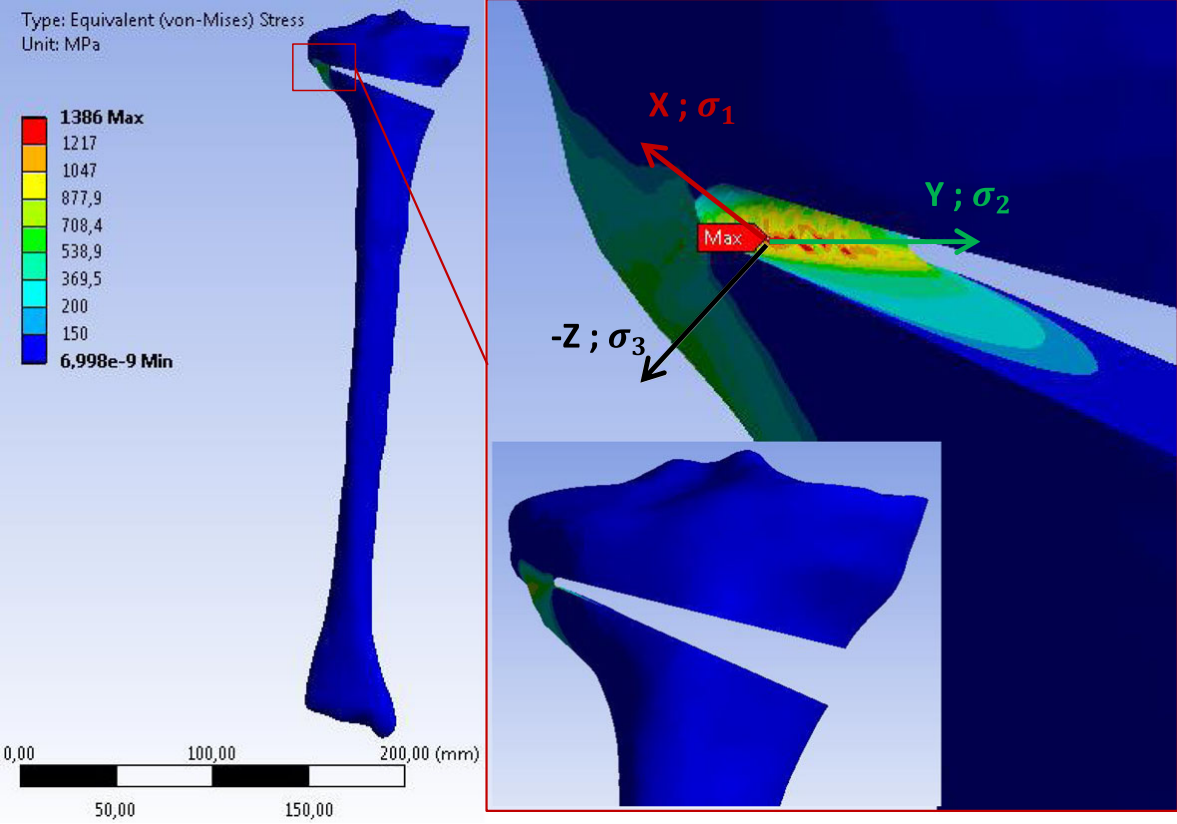
Equivalent (von-Mises) stress in the specimen SNH obtained with 3D linear FEA: Only the result for case study H is represented. The stress values correspond to an opening angle of 10°. The specimen has been slightly rotated in the zoom-view on the right to show the location of the reference point where the equivalent (von-Mises) stress was maximum

**Fig. 11:**
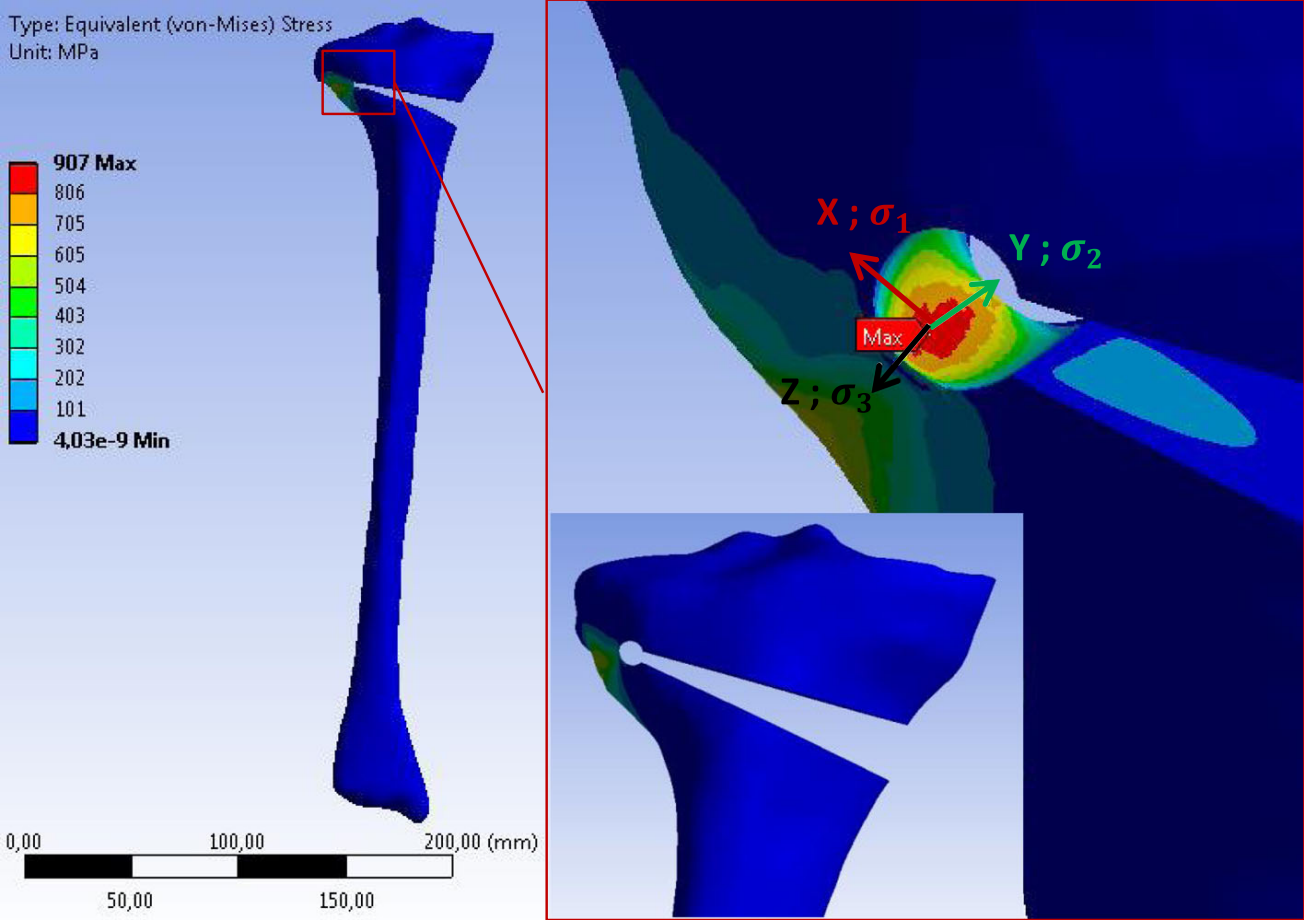
Equivalent (von-Mises) stress in the specimen SWHE obtained with 3D linear FEA: Only the result for case study H is represented. The stress values correspond to an opening angle of 10°. The specimen has been slightly rotated in the zoom-view on the right to show the location of the reference point where the equivalent (von-Mises) stress was maximum

**Fig. 12:**
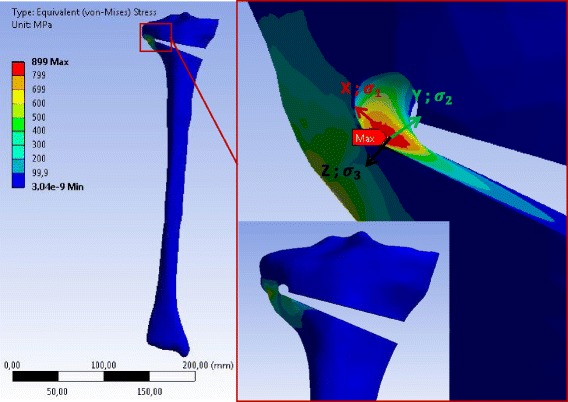
Equivalent (von-Mises) stress in the specimen SWHA obtained with 3D linear FEA: Only the result for case study H is represented. The stress values correspond to an opening angle of 10°. The specimen has been slightly rotated in the zoom-view on the right to show the location of the reference point where the equivalent (von-Mises) stress was maximum

The stress distributions after performing static linear analysis of opening the osteotomy cut by 10° were similar for any specimen independently of the case study (Table 3). The equivalent (von-Mises) stresses obtained for the case H are depicted in Fig. 9 for the 2D-analysis and in Figs. 10, 11 and 12 for the 3D-analysis.

The maximum values of the equivalent (von-Mises) and principal stresses for the 2D linear FEA are summarized in Table 5.

**Table 5:** Stresses at the reference point of the different specimens after the 2D linear FEA

Cases	Specimens	Max. equiv. stress *σ*_*eq*,*max*_(MPa)	Max. princ. stress *σ*_1_(MPa)	Mid. princ. stress *σ*_2_(MPa)	Min. princ. stress *σ*_3_(MPa)
H	SNH	1548	1548	0	0
	SWHE	973	973	0	0
	SWHA	986	986	0	0
M	SNH	1275	1275	0	0
	SWHE	801	801	0	0
	SWHA	812	812	0	0
L	SNH	1366	1366	0	0
	SWHE	858	858	0	0
	SWHA	870	870	0	0

The principal stresses *σ*_2_ and *σ*_3_ were zero due to the fact that plane stress state was assumed and the reference point was located on an unloaded border

For the 2D FEA the maximum equivalent (von-Mises) stress was located at the tip of the osteotomy cut of the specimen SNH. The corresponding value was smaller for specimens with a drill hole at the end of the osteotomy cut (specimens SWHE and SWHA in Table 5). The specimen SWHE showed the smallest maximum. This means that drilling a hole at the end of the osteotomy cut reduces the stress in the contralateral cortical.

The middle and the minimal principal stresses were zero, meaning that the stress at the reference point was uniaxial in the first principal direction (vector X in the enlargement of Fig. 9). The maximum principal stresses were all positive, indicating that the stress state at the reference point was tension (Table 5).

According to the linear 3D FEA the maximum equivalent (von-Mises) stress was located at the tip of the notch more on the inside of the osteotomy cut. The maximum values of the principal equivalent stresses are summarised in Table 6. As observed for the 2D analysis, the maximum was smaller in the cases with a drill hole, while the differences between SWHA and SWHE were very small and not relevant. The maximum and the middle principal stresses at the reference points were positive, indicating that the stress state at the reference points was two-dimensional tension. The minimum principal stress was zero, meaning that the stress-state at the reference point was plane (vector X and Y in the enlargement of Figs. 10, 11 and 12).

**Table 6:** Stresses at the reference point of the different specimens after 3D linear FEA

Cases	Specimens	Max. equiv. stress *σ*_*eq*,*max*_(MPa)	Max. princ. stress *σ*_1_(MPa)	Mid. princ. stress *σ*_2_(MPa)	Min. princ. stress *σ*_3_(MPa)
H	SNH	1386	1548	421	0
	SWHE	907	1003	239	0
	SWHA	899	996	242	0
M	SNH	1145	1279	349	0
	SWHE	747	826	196	0
	SWHA	741	820	200	0
L	SNH	1228	1371	373	0
	SWHE	801	885	210	0
	SWHA	793	879	214	0

The minimum principal stress *σ*_3_ was zero because the reference point was located on an unloaded border. It means that the stress-state at the reference point was plane

#### Factor *K*_*p*_ characterizing the load bearing reserve up to the PLL

**Fig. 13:**
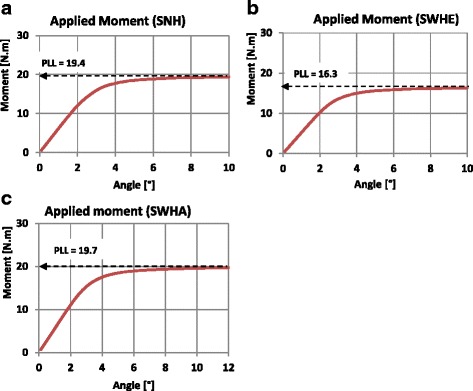
Determination of PLL: Nonlinear load–displacement diagrams. Only the curves from the 3D FEA (case study H) are shown here. **a** Specimen SNH, **b** Specimen SWHE, **c** Specimen SWHA

The values of ELL were estimated with the previous linear FEA that were used to determine the local stresses at the reference point. Nonlinear 2D and 3D FEA were performed using the different bilinear material models of the case studies H, M and L of Fig. 7. The osteotomy cut was opened until a horizontal tangent in the load–displacement diagram was reached (PLL), as shown in the examples depicted in Fig. 13. The factor *K*_*p*_ was calculated according to Eq. (7) and summarised with the values of the PLL and ELL in Table 7 for the 2D FEA and Table 8 for the 3D FEA.

**Table 7:** Factor *K*_*p*_ from 2D finite element analysis

Cases	Specimens	ELL (Nm)	PLL (Nm)	*K*_*p*_
H	SNH	2.6	8	3.2
	SWHE	3.5	8	2.3
	SWHA	3.5	8	2.3
M	SNH	2.1	6.5	3.2
	SWHE	2.8	6.5	2.3
	SWHA	2.8	6.5	2.3
L	SNH	1.7	5.1	3.2
	SWHE	2.2	5	2.3
	SWHA	2.2	5.1	2.3

**Table 8:** Factor *K*_*p*_ from 3D finite element analysis

Cases	Specimens	ELL (Nm)	PLL (Nm)	*K*_*p*_
H	SNH	5.1	19.4	3.8
	SWHE	6.1	16.3	2.7
	SWHA	7.2	19.6	2.7
M	SNH	3.6	16.4	4.6
	SWHE	4.3	13.6	3.2
	SWHA	5.1	17.5	3.5
L	SNH	3.4	13	3.9
	SWHE	4	10.8	2.7
	SWHA	4.7	14.1	3

The factor *K*_*p*_ was higher for specimens SNH, because the yield stress in its contralateral cortical was reached at the lower load level in linear FEA compared to the specimens with drill holes.

#### The degree of multiaxiality *h*, the critical value of total strain *ε*_*ertr*_ and the factor $\left(\sqrt{\  E{\varepsilon}_{e rtr}/{R}_e}\right)$

The values of the degree of multiaxiality *h* and the critical total strain *ε*_*ertr*_ calculated in accordance with Eqs. (3) and (5) respectively, considering the material parameters of Table 4, are summarised together with the values of the factor $\left(\sqrt{E{\varepsilon}_{e rtr}/{R}_e}\right)$ in Table 9 (for the cortical bone considered as semi-ductile material) and in Table 10 (for the cortical bone considered as ductile material).

**Table 9:** Degree of multiaxiality *h*, critical value of total strain *ε*_*ertr*_ and factor $\left(\sqrt{E{\varepsilon}_{e rtr}/{R}_e}\right)$

Cases	Specimens	2D FEA			3D FEA		
		*h*	*ε*_*ertr*_ [%]	$\sqrt{E{\varepsilon}_{e rtr}/{R}_e}$	*h*	*ε*_*ertr*_ [%]	$\sqrt{E{\varepsilon}_{e rtr}/{R}_e}$
H	SNH	0.33	1.2	1.36	0.47	0.8	1.1
	SWHE	0.33	1.2	1.36	0.46	0.8	1.1
	SWHA	0.33	1.2	1.36	0.46	0.8	1.1
M	SNH	0.33	1.5	1.61	0.47	0.8	1.17
	SWHE	0.33	1.5	1.61	0.46	0.8	1.2
	SWHA	0.33	1.5	1.61	0.46	0.8	1.19
L	SNH	0.33	0.6	1.07	0.47	0.5	1
	SWHE	0.33	0.6	1.07	0.46	0.5	1.01
	SWHA	0.33	0.6	1.07	0.46	0.5	1.01

These values were obtained for the cortical bone considered as semi-ductile material for 2D and 3D FEA

**Table 10:** Degree of multiaxiality *h*, critical value of total strain *ε*_*ertr*_ and factor $\left(\sqrt{E{\varepsilon}_{e rtr}/{R}_e}\right)$

Cases	Specimens	2D FEA			3D FEA		
		*h*	*ε*_*ertr*_ [%]	$\sqrt{E{\varepsilon}_{e rtr}/{R}_e}$	*h*	*ε*_*ertr*_ [%]	$\sqrt{E{\varepsilon}_{e rtr}/{R}_e}$
H	SNH	0.33	0.6	3	0.47	3.2	2.2
	SWHE	0.33	0.6	3	0.46	3.5	2.28
	SWHA	0.33	0.6	3	0.46	3.5	2.27
M	SNH	0.33	0.6	3.24	0.47	3.2	1.37
	SWHE	0.33	0.6	3.24	0.46	3.5	2.46
	SWHA	0.33	0.6	3.24	0.46	3.4	2.45
L	SNH	0.33	0.6	3.38	0.47	3.2	2.48
	SWHE	0.33	0.6	3.39	0.46	3.5	2.58
	SWHA	0.33	0.6	3.39	0.46	3.4	2.56

These values were obtained for the cortical bone considered as ductile material for 2D and 3D FEA

#### The relevant factor *n*_*pl*_, the specimen static strength *σ*_*SK*_ and the critical opening angle α_crit_

Table 11 (cortical bone considered as semi-ductile) and Table 12 (cortical bone considered as ductile) list the values of the relevant factor *n*_*pl*_ calculated according to Eq. (2) by considering the values of the notch factor *K*_*p*_ (Table 7 and Table 8) and the factor $\sqrt{E{\varepsilon}_{e rtr}/{R}_e}$ (Table 9 and Table 10), the static strength *σ*_*SK*_ calculated in accordance with Eq. (1) and the critical opening angle α_crit_ reached in the linear finite elements calculations when the relevant combined stress according to Eq. (8) reached *σ*_*SK*_.

**Table 11:** Static strength and critical angles of the specimens with the cortical bone considered as semi-ductile

Cases	Specimens	2D FEA			3D FEA		
		*n*_*pl*_	*σ*_*SK*_ [MPa]	α_crit_ [°]	*n*_*pl*_	*σ*_*SK*_ [MPa]	α_crit_ [°]
H	SNH	1.36	155	1	1.1	123	0.9
	SWHE	1.36	155	1.6	1.1	125	1.4
	SWHA	1.36	155	1.6	1.1	125	1.3
M	SNH	1.61	129	1	1.17	93	0.7
	SWHE	1.61	129	1.6	1.2	96	1.2
	SWHA	1.61	129	1.6	1.19	96	1.2
L	SNH	1.07	84	0.6	1	79	0.6
	SWHE	1.07	84	1	1.01	79	0.9
	SWHA	1.07	84	1	1.01	79	0.9

**Table 12:** Static strength and critical angles of the specimens with the cortical bone considered as ductile

Cases	Specimens	2D FEA			3D FEA		
		*n*_*pl*_	*σ*_*SK*_ [MPa]	α_crit_ [°]	*n*_*pl*_	*σ*_*SK*_ [MPa]	α_crit_ [°]
H	SNH	3	341	2.2	2.2	251	1.8
	SWHE	2.3	264	2.7	2.28	260	2.9
	SWHA	2.3	262	2.7	2.27	259	2.9
M	SNH	3.1	253	2	1.37	190	1.7
	SWHE	2.3	185	2.3	2.46	197	2.6
	SWHA	2.3	184	2.3	2.45	196	2.6
L	SNH	3.1	249	1.8	2.48	194	1.6
	SWHE	2.3	182	2.1	2.58	201	2.5
	SWHA	2.3	181	2.1	2.56	200	2.5

For any case (H, M or L) the critical angle was higher for specimens with drill hole (SWHE and SWHA) than for specimens without. For all specimens the critical angle decreases when we go from case study M (young patients) to case study L (old patients) with the lower ultimate strength.

**Fig. 14:**
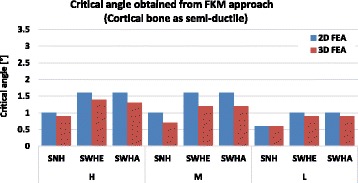
Critical angle obtained using FKM approach (Cortical bone as semi-ductile): The critical angle was the angle obtained when the equivalent stress in the specimen reached the assessed static strength. The higher critical angles were obtained for specimens with drilled holes (SWHE and SWHA)

**Fig. 15:**
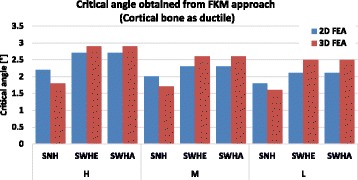
Critical angle obtained using FKM approach (Cortical bone as ductile): The critical angle was the angle obtained when the equivalent stress in the specimen reached the assessed static strength. The higher critical angles were obtained for specimens with drilled holes (SWHE and SWHA)

Figures 14 and 15 recapitulate the critical angles obtained by using the FKM approach assuming semi-ductile and ductile material behaviour respectively.

The differences between the 2- and the 3-dimensional finite element analyses were less than 10% on average and hence, were not important. The constitutive material behaviour however is very important, as the critical opening angle α_crit_ was 1.1° on average for semi-ductile (Table 11 and Fig. 14) and 2.3° on average for ductile material (Table 12 and Fig. 15). Both angles were still quite small compared to real angles during surgery.

### Results using the strain approach

**Fig. 16:**
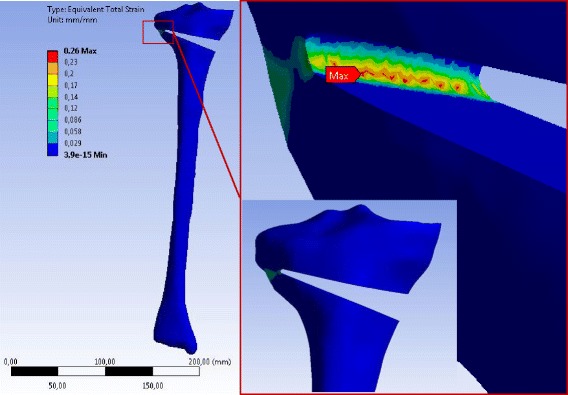
Equivalent total strain (von-Mises) in the specimen SNH for material case L: The total strain was calculated by nonlinear 3D FEA. The osteotomy gap was opened by 10°. The specimen has been slightly rotated in the enlargement picture

**Fig. 17:**
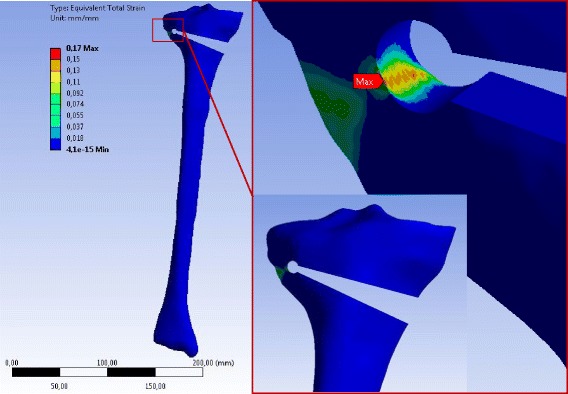
Equivalent total strain (von-Mises) in the specimen SWHE for material case L: The total strain was calculated by nonlinear 3D FEA. The osteotomy gap is opened by 10°. The specimen has been slightly rotated in the enlargement picture

**Fig. 18:**
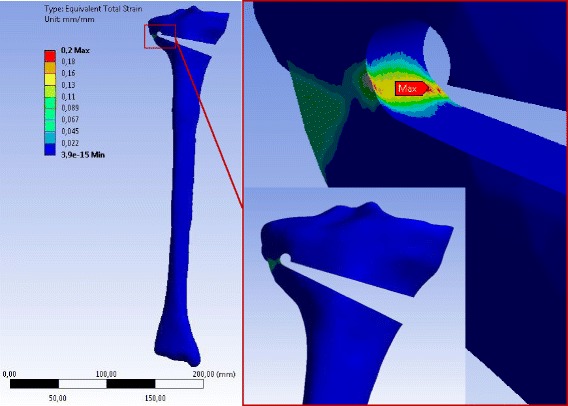
Equivalent total strain (von-Mises) in the specimen SWHA for material case L: The total strain was calculated by nonlinear 3D FEA with an opening of the osteotomy gap by 10°. The specimen has been slightly rotated in the enlargement picture

The maximum equivalent total strain for all specimens was located in the contralateral cortex when an opening the osteotomy gap was performed. Figures 16, 17 and 18 show for instance the total equivalent strain in the specimens for case study L, i.e. patients older than 80 years. The cortical bone was considered to behave nonlinearly according to the stress–strain curves in Fig. 8.

**Fig. 19:**
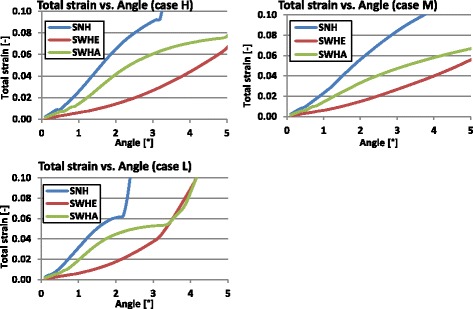
Total strain versus opening angle calculated with nonlinear 2D FEA: The total strain increased faster for the specimen with no hole (SNH)

**Fig. 20:**
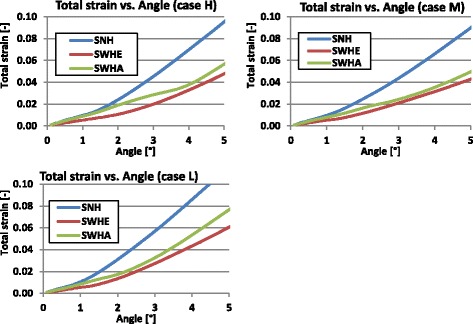
Total strain versus opening angle calculated with nonlinear 3D FEA: The total strain increased faster for the specimen with no hole (SNH)

The following graphs shown in Figs. 19 and 20 indicate the total strain in function of the opening angle for the 2D FEA and the 3D FEA respectively.

Studying Figs. 19 and 20 it turned out that the total strain obtained with 2D FEA increased faster than in 3D FEA, which resulted in smaller critical angles using the 2D FEA. Furthermore, the specimens without a hole (SNH) yielded more quickly than those with a hole (SWHE, SWHA).

**Fig. 21:**
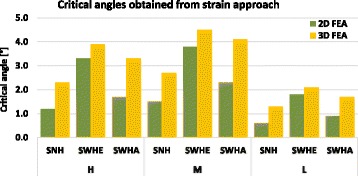
Calculated critical angles of the specimens with failure criterion total strain ≤ A: The higher critical angles were obtained for specimens with drilled holes and the highest for the specimen SWHE

Table 13 summarises the critical angles obtained when the total strain reached the elongation at break *A*. Figure 21 gives a recapitulative comparative diagram of all determined critical angles.

**Table 13:** Calculated critical angles based on the strain approach

Cases	Specimens	Critical angle α_crit_ [°]	
		2D FEA	3D FEA
H	SNH	1.2	2.3
	SWHE	3.3	3.9
	SWHA	1.7	3.3
M	SNH	1.5	2.7
	SWHE	3.8	4.5
	SWHA	2.3	4.1
L	SNH	0.6	1.3
	SWHE	1.8	2.1
	SWHA	0.9	1.7

For any specimen the critical angle decreased with the elongation at break corresponding to an increasing of the age of the patient increases (Fig. 21). The highest values were obtained for case M, i.e. young adults with highest elongation at break *A* = 3.7%. The elongation at break *A* (i.e. the ductility of the material) was more important than its strength, as can be seen by comparing case H (Tensile strength *R*_*e*_ = 114 *MPa* and *A* = 3.1%) and case M (Tensile strength *R*_*e*_ = 80 *MPa* and *A* = 3.7%). The maximal critical angle of 4.5°obtained for young patients (case M) is approximately the double of that for old patients (case L).

**Fig. 22:**
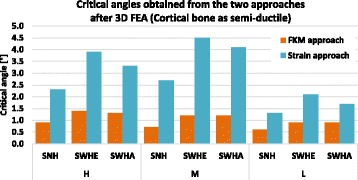
Critical angles of the specimens: The values obtained with strain approach were higher compared to those obtained with FKM approach. The highest critical angle (4.5°) was obtained for specimen SWHE considering the material case M (young patients)

The FKM approach with semi-ductile material (*A* < 6%) and the strain approach with the more realistic 3D FEA were chosen for final comparison. The critical angles are shown in Fig. 22.

It became clear that FKM approach is more conservative, which appeared natural for a norm intended for strength assessment. Furthermore, all correction angles above 4.5° would lead to crack initiation in the contralateral cortex, even if the more generous strain approach was chosen for design criterion.

## Discussion

One key finding of the study was that drilling a hole at the end of the osteotomy reduces the stresses in the contralateral cortex and increases the opening angle prior to cracking by maximal 65%. The other key finding was that all calculated absolute angles prior to crack initiation were below 5° and hence small, leading to the presumption that at least cracking, but probably also full rupture, takes place in nearly all cases with correction angles above 5°.

Three different specimens have been used: a specimen without a drill hole (SNH), a specimen with a drill hole centrically in line with, and at the end of, the osteotomy (SWHE) and a specimen with the drill hole located at the proximal end of the osteotomy (SWHA). The simulated surgical technique and the geometric characteristics of the osteotomy height, obliquity and minimal distance to the opposite cortex was the same for all specimens.

Three material cases have been considered to reflect the physical variety depending mainly on the age of the patients. Case H was designed with the highest mean strength values found in literature. For cases M and L the material parameters used were those of young (20–29 years) and old (more than 80 years) patients respectively. While using the FKM approach, the cortical bone was considered firstly as semi-ductile and secondly as ductile following the differentiation proposed by the FKM-guidelines (Rennert et al. 2012). The ductile material assumption was made only to highlight this influence on the assessed strength.

2D and 3D FEA were performed. The load applied consisted of a continuous opening (10°) of the osteotomy gap. Regardless of the analysis type, the stress concentration was located in the zone near to the tip of the osteotomy cut. The maximum equivalent (von-Mises) stress was smaller for specimens with a drill hole at the end of the osteotomy cut.

The reference stress at the hot-spot was comparable for specimens SWHE and SWHA (Table 5 and Table 6). This means that any of the SWHE and SWHA specimens can be considered in order to reduce the stress in the opposite cortex. The maximum value of the critical opening angle was obtained for specimen SWHE, meaning that drilling the hole as an extension of the osteotomy cut is optimal, though the differences to SWHA are small.

The specimens with a hole (SWHE, SWHA) yielded more slowly than those with no hole (SNH). This means that the hole enlarges the volume of highly charged material and hence leads to a smaller strain for a given opening angle or in larger values for the critical angle.

For all specimens the critical angle leading to crack initiation decreased when the patient age increased. The absolute difference between the critical angle of the specimens with a drill hole and the specimens with no hole decreased with the patient age. This means that drilling a hole in order to reduce the critical angle is more advantageous in case of young patients. This is correlated to the fact that the cortical bone of old patients is less ductile. By comparing the results obtained with the FKM approach of semi-ductile (Fig. 14) and of ductile (Fig. 15) material it becomes clear that ductility is the decisive parameter. This fact was also observed by using the strain approach, where maximum values for the critical angles were obtained for case study M, where elongation at break was highest (Fig. 6).

In specimens with larger correction angles exceeding approximately 4.5° cracks are initiated and material segregation is started even with relative ductile bone behaviour, i.e. in case of younger patients. Large correction angles hence always lead at least to micro-cracking in the zone of the tip of the osteotomy cut. The clinical relevance of micro-cracking in the zone of the tip of the osteotomy cut was not investigated here. In case of older people with reduced ductility and strength of the cortical bone, the cracking starts earlier, i.e. at smaller angles.

The assessment was based on crack initiation only and hence complete dislocation was not investigated here. But as the wall thickness from the drilled hole to the outer surface of the bone is small, it can be concluded by simple engineering reasoning that complete rupture of the opposite cortex follows soon after crack initiation. These data suggest that the hinge pin offers no relevant benefit for the treatment of malalignment since the need for correction in patients with varus alignment is frequently above 8° of correction. It is well documented that the risk of revarisation in the presence of the opposite cortex fracture increases with every degree of valgus correction (Pape et al. 2004a, 2004b). Obviously, the current study suggests that the cortex-preserving hinge pin effect is absent in larger corrections exceeding 5°. This observation is confirmed in an experimental study by Reyle et al. (2017), which used human cadaver tibial bone, artificial Synbones and Sawbones to investigate the fracture of the lateral hinge in an open-wedge HTO with and without a hinge pin.

Seemingly, it is difficult to determine the validity of biomechanical experiments done in the absence of muscular support and power. In this laboratory setting, the definition of a fracture of the opposite cortex followed an “all-or-nothing principle” due to the used underlying numerical model. Moreover, differences between the critical angles are small and it could be concluded that there is no significant difference between the specimens. The cortical bone has been considered as homogenous, isotropic linear elastic or elastic-ideal plastic, and the results obtained are based only on numerical calculations in ideal situations, which should eventually be completed with experimental studies. Furthermore, the method used for the FKM approach is normally dedicated to steel, aluminium and their alloys. Considering the tibia head as composed only of cortical bone constitutes another limitation of the present study. Hence, caution is required when transferring the results of this study to clinical settings.

In clinical practice, the definition of a fracture of the opposite cortex is less precise unless a true dislocation and separation of the two cortices are apparent on intraoperative fluoros-copy. (Staubli 2008) found in her significant number of osteotomy patients multiple small fissures, bone bruises without an apparent fracture on intraoperative fluoroscopic images.

Obviously, the capacity for maintaining bony stability in the presence of (multiple) cortex fissures can vary significantly among patients and bone stability does not allow an “all-or-nothing principle”. Nevertheless, the data of the present study indicate that a correctly placed hinge pin at the end of the horizontal and ascending tibial osteotomy does not prevent opposite cortex fracture in larger corrections. The data suggest that bone ductility is an important factor for the crack initiation and hence integrity of opposite cortex, especially for higher corrections.

## Conclusions

The current study suggests that the drill hole (hinge pin) at the end of the horizontal osteotomy reduces the stresses in the contralateral cortex and hence increases the critical opening angle prior to cracking of the opposite cortex in specimen with small correction angles. But the difference from having a drill hole or not is not so significant, especially for older patients. The ductility of the cortical bone is the decisive parameter for the calculated critical opening angle. Transferred to clinical practice, these data suggest that the hinge pin is an asset, but not the decisive parameter, and it cannot avoid cracking and rupture of the opposite cortex in cases of larger correction angles.

## Abbreviations

2D FEA: Two dimensional finite element analysis
2D: Two dimensional
3D FEA: Three dimensional finite element analysis
3D: Three dimensional
AP: Anterior posterior
COWHTO: Closing wedge high tibial osteotomy
ELL: Elastic limit load
FEA: Finite element analysis
FKM: “Forschungskuratorium Maschinenbau”
OA: Osteoarthritis
OWHTO: Opening wedge high tibial osteotomy
PLL: Plastic limit load
SNH: Specimen without hole
SWHA: Specimen with the drill hole at the end of the osteotomy a little bit above
SWHE: Specimen with the drill hole at the end of the osteotomy cut as an extension
